# Risk assessment of labial bone perforation in the anterior mandibular region: a virtual immediate implant placement study

**DOI:** 10.1186/s40729-021-00351-w

**Published:** 2021-07-26

**Authors:** Yi-Wen Cathy Tsai, Ren-Yeong Huang, Chia-Dan Cheng, Wan-Chien Cheng, David L. Cochran, Thomas T. Nguyen, Yi-Shing Shieh, Fu-Gong Lin, Cheng-En Sung

**Affiliations:** 1 Department of Periodontology, School of Dentistry, Tri-Service General Hospital and National Defense Medical Center, Taipei, Taiwan; 2 Department of Periodontics, The University of Texas Health Science Center at San Antonio, San Antonio, TX USA; 3 Division of Periodontics, Faculty of Dentistry, McGill University, Montreal, QC Canada; 4 Department of Oral Diagnosis and Pathology, School of Dentistry, Tri-Service General Hospital and National Defense Medical Center, Taipei, Taiwan; 5 School of Public Health, National Defense Medical Center, Taipei, Taiwan; 6 Department of Optometry, University of Kang Ning, Taipei, Taiwan

**Keywords:** Cone beam computed tomography, Mandible, Classification, Risk assessment, Dental implantation, Labial bone perforation

## Abstract

**Background:**

This study investigated the prevalence of labial bone perforation (LBP) related to the associated anatomic factors in anterior mandibular region using a virtual immediate implant placement procedure.

**Methods:**

Series qualified CBCT images of 149 participants (894 teeth) were selected to analyze the assigned anatomical parameters, including concavity depth, concavity angle, torque, and deep bone thickness. Four classes of crestal and radicular dentoalveolar bone phenotypes (CRDAPs) of mandibular anterior teeth were categorized according to the thickness of dentoalveolar bone at both crestal and radicular zones. Data were adjusted for categorical (gender and CRDAP) and continuous (age, cavity angle, cavity depth, and deep bone thickness) variables using a multivariable logistic regression analysis with generalized estimating equation method.

**Results:**

The overall probability of LBP after virtual implant placement was 21.6%. There is statistically significant higher prevalence of LBP at canine (28.5%) and CRDAP class II (29.2%) regions (*p* < 0.001). After adjusting confounding variables, CRDAP class II and class IV regions are more likely to have LBP when compared with CRDAP class I (control) regions (*p* < 0.01). The risk of LBP at canine site is 6.31 times more likely than at the central incisor (control) (*p* < 0.01).

**Conclusions:**

Using a virtual immediate implant placement technique, the prevalence of LBP is significantly higher at the mandibular canine site and thin radicular dentoalveolar phenotype in the anterior mandibular region.

**Supplementary Information:**

The online version contains supplementary material available at 10.1186/s40729-021-00351-w.

## Introduction

Currently, placing an implant immediately into an extraction socket is still believed to be a technique-sensitive procedure, even though high survival and success rates of immediate implant treatment have been reported [1–3]. Surgical complications related to immediate implant placement may compromise the outcome and even result in life-threatening events [4–9]. Therefore, preventive and precautionary risk assessment should be taken before implant placement to reduce the probability of surgical-related risks and achieve a satisfactory outcome. The operator should conduct comprehensive preoperation risk assessments and apply thorough knowledge of the surgical zone’s anatomical features when deciding the optimal position and angulation of implants [4–10].

With regard to anatomic risks during immediate implant placement, many regions of interest have been studied previously [10–15]. For the posterior mandible region, the existence of lingual concavity, perforation of the lingual cortical plate, and geometric features of inferior alveolar nerve in the mandible limit the immediate implant placement procedure [7, 10, 12, 14–16]. In the anterior maxilla region, labial bone deficiency, and the particular relationship between teeth and alveolar process may increase the risk of perforation of the facial cortical bone [10, 11, 17]. Consequently, soft tissue recession for the thin labial bone thickness of anterior maxillary teeth potentially compromised the esthetic outcomes [13, 18, 19]. Therefore, adequate knowledge of prosthetically driven treatment plan should be applied to prevent vital tissue damage, such as cortical bone perforation.

Although numerous studies regarding the decreased anatomic risks of immediate implant have been proposed, cases about labial bone perforation (LBP) in anterior mandibular region compromising the implant survival have still been reported, but the assessment of risk of LBP in this region is limited [20, 21]. Therefore, the purpose of this study was to investigate the prevalence of crestal and radicular dentoalveolar bone phenotype (CRDAP) and evaluate anatomic factors contributing to the prospect of LBP in the anterior mandibular region using a virtual immediate implant placement in cone beam computed tomography (CBCT) images.

## Methods

### Database confidentiality, image acquisition, and retrieving

In this retrospective observational study, consecutive CBCT images were retrieved and investigated from a CBCT database possessed by the Department of Dentistry, Tri-Service General Hospital, National Defense Medical Center, Taipei, Taiwan (from November 2013 to December 2016). All images were not taken specifically for this project. The qualified images (subjects and teeth) that met the inclusion and exclusion criteria were selected for analysis (Supplemental Figure 1 and Supplemental Table 1). The protocol used in this study was reviewed and approved by the Ethics Committee and Institutional Review Board of Tri-Service General Hospital, National Defense Medical Center (TSGHIRB No. 2-102-05-064).

The CBCT machine (NewTom 5G; QR, Verona, Italy) was operated by board-certified radiologist following the standard manufacturer’s settings as previously described [10, 12]. The skull orientation and region of interest were taken according to previous studies [10, 15, 22]. The maxilla was bilaterally symmetric and the occlusal plane, either in the frontal or sagittal view, was parallel to the ground (Supplemental Figure 2A). The acquired CBCT images were saved in a Digital Imaging and Communications in Medicine (DICOM) format, and these data were confidentially protected.

### Inclusion and exclusion criteria of selected CBCT images

The CBCT images had to fulfill the following inclusion and exclusion criteria as previously described [10, 23]. The inclusion criteria had to be as follows [10, 23]:

• Permanent mandibular central incisor, lateral incisor, or canine had to be fully erupted with fully formed apexes;

• Each examined tooth had to be normally positioned with normal alignment, with a harmonious incisal line across the mandibular anterior teeth;

• Opposing maxillary teeth had to be present to provide information for optimal implant angulation and inclination;

Subjects were excluded if images showed [10, 23]:

• Bone screws and plates for surgical treatments, or any grafted materials;

• Preexisting alveolar bone destruction, perforation, dehiscence, or a combination of these caused by periodontal disease or traumatic injury around the investigated region;

• Supernumerary or impacted tooth;

• A pathological lesion, or evident root resorption;

• Incompletely formed apex;

• Dental misalignment, or preexisting dental implant;

• Signs of prosthodontic treatment, root canal treatments, and/or apical surgery;

• Obscurity or distortion due to scattering, or beam-hardening artifact reasons.

### Assessment and classification of the crestal and radicular dentoalveolar phenotype (CRDAP) of anterior mandibular teeth

**Fig. 1:**
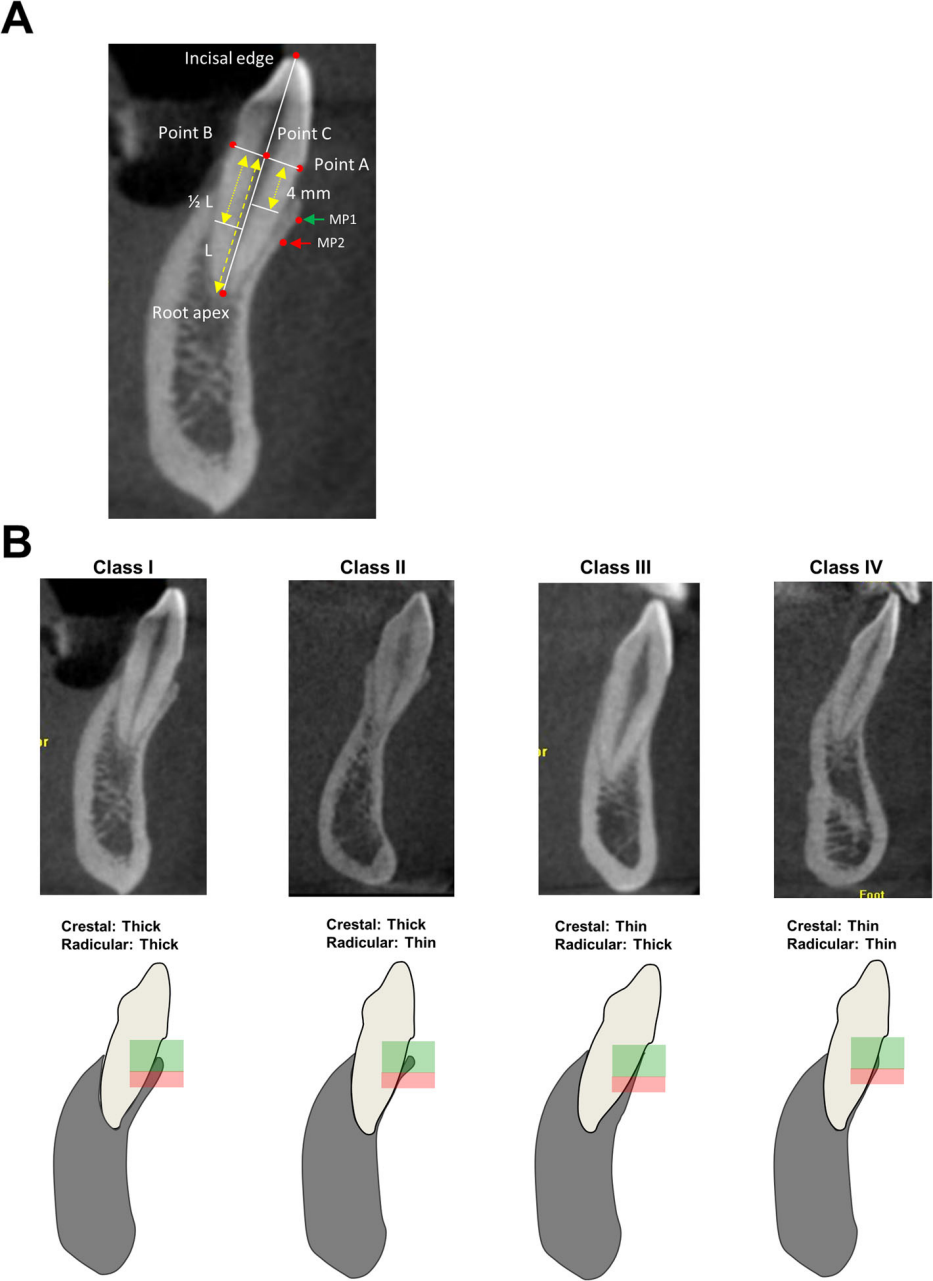
Landmarks and classification of the crestal and radicular dentoalveolar phenotype (CRDAP) of mandibular anterior teeth in relation to the anterior mandibular osseous housing in sagittal radiographic images. **a** Landmarks in the sagittal radiographic images. **b** Radiographic images of four types of CRDAP (upper panel). Schematic illustrations of four types of CRDAP (class I, II, III, and IV) (lower panel), which categorized by the alveolar bone thickness at crestal (green color) and radicular (red color) region

The following reference points and landmarks were defined on reconstructed images by viewing a sagittal-sectioned image of the region of interest and the center section of each investigated tooth (Fig. 1a) [24].

• Point A and Point B: the mid-facial and mid-lingual CEJ of the tooth, respectively;

• Point C: the intersection point of line A-B and line connecting the incisal edge and root apex;

• Length of the root (L): the distance between root apex and the Point C;

• Measuring point 1 (MP1): the point located 4 mm apical to the CEJ on facial root surface;

• Measuring point 2 (MP2): the point located middle of the root on buccal root surface.

Two dentoalveolar zones, “crestal zone” and “radicular zone”, were defined with minor modification to fit the purpose of this study accordingly (Fig. 1b) [25]. The crestal zone of dentoalveolar bone was defined as the region from the facial CEJ extending to a point 4-mm apical (MP1). The radicular zone was dependent upon individual root length and was defined as the region from MP1 to the MP2 (Fig. 1). The thickness of facial dentoalveolar bone on both crestal and radicular zones could be determined as either thick or thin phenotype [25]. For thick phenotypes, the facial bone thickness was defined as ≥ 1 mm, whereas, for thin phenotypes, the thickness was < 1 mm.

Four classes of crestal and radicular dentoalveolar phenotype (CRDAP) of mandibular anterior teeth were categorized according to the thickness of dentoalveolar bone at both crestal and radicular zones at the tooth level (Fig. 1b) [25]:

• Class I: both the crestal and radicular dentoalveolar zones were thick phenotype.

• Class II: the crestal zone was thick, but the radicular zone was thin phenotype

• Class III: the radicular zone was thick, but the crestal zone was thin phenotype

• Class IV: both the crestal and radicular dentoalveolar zones were thin

phenotype.

### Measurements of morphological features of mandibular anterior region

**Fig. 2:**
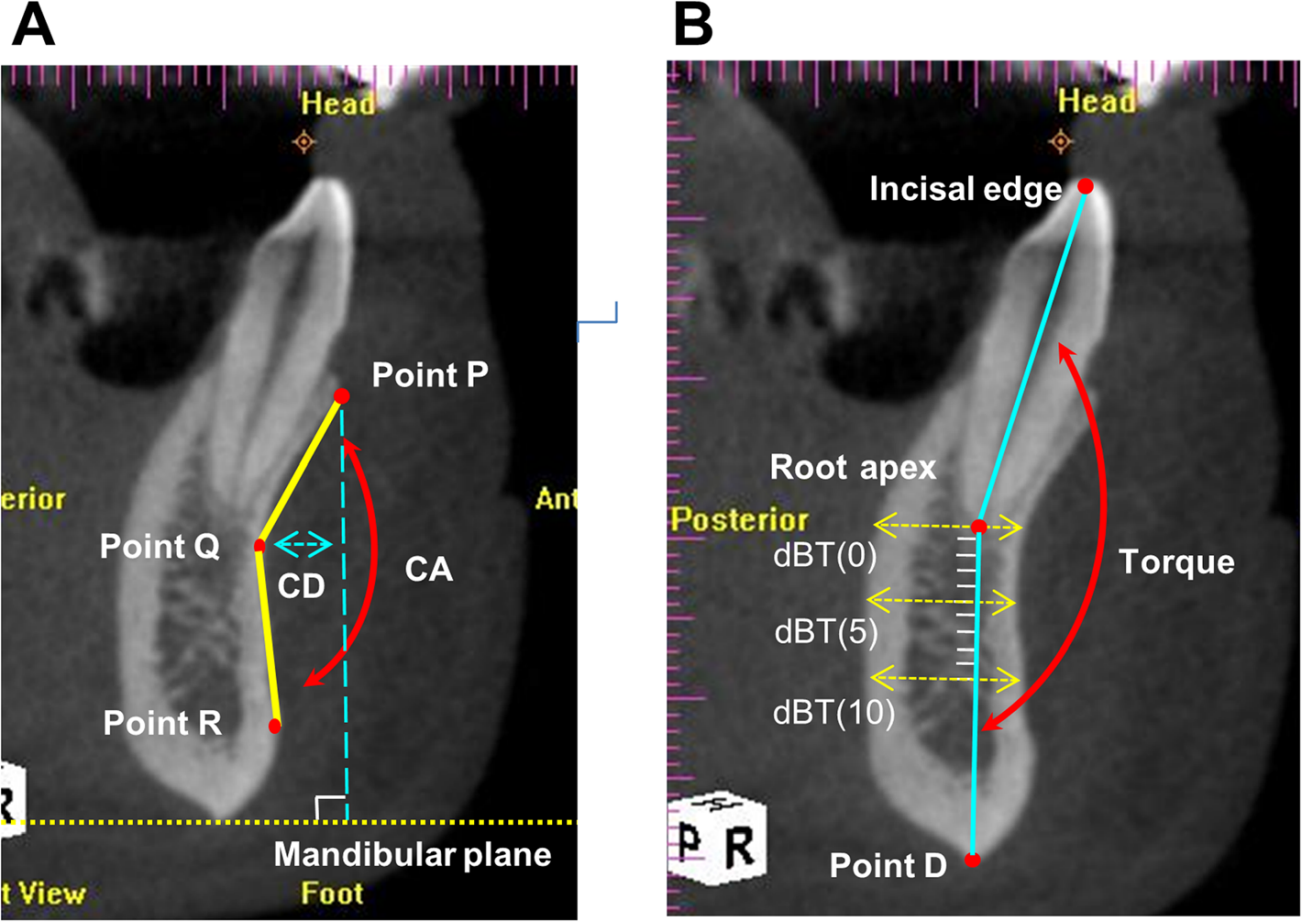
Anatomical parameters regarding labial bone perforation (LBP) of the anterior mandibular region in sagittal radiographic images. **a** Landmarks and anatomical parameters of the concavity depth (CD), and concavity angle (CA). **b** Landmarks and anatomical parameters of the torque (T), and deep bone thickness (dBT). Each measurement line of dBT was also perpendicular to the mandibular long axis, and then the distance between the facial and lingual outline of the bone was measured

The following morphologic and dimensional parameters of the mandibular teeth and alveolar ridge were measured accordingly (Fig. 2) [10, 17, 26].

• Concavity depth (CD): the distance between the deepest point of the facial bone plate (point Q) and a vertical reference line perpendicular to the mandibular plane, passing through the most external point of the labial plate (point P) (Fig. 2a).

• Concavity angle (CA): the angulation between line Q-P (that is, the line connecting points Q and P, with point P defined as the most external point of the labial plate) and line Q-R (that is, the line connecting points Q and R, with point R defined as the most external point of the labial plate inferior to point Q, and relatively lower than the apex of the tooth along the apico-coronal continuum) (Fig. 2a).

• Torque (T): the angle formed between the long axis of a tooth (that is, the line connecting incisal edge and root apex of the tooth) and long axis of the mandible, connecting from root apex to point D (that is, the lowest point of the mandible) (Fig. 2b) [26].

• Deep bone thickness (dBT): mandibular deep bone thickness was measured at 0, 5, and 10 mm from tooth root apex and along the long axis of the mandible (that is, the line connecting the tooth apex and point D), presented as dBT (0), dBT (5), and dBT (10), respectively (Fig. 2b). Each measurement line was also perpendicular to the mandibular long axis, then the distance between the facial and lingual outline of the bone was measured [26].

### Virtual implant selection, placement, and definition of labial bone perforation (LBP)

The root form taper-designed dental implants were selected from an implant database available in the CBCT software as previously described [10, 12, 16]. The diameter of dental implants was determined by mimicking the corresponding size of each investigated tooth root, 3.0 mm for central and lateral incisors and 4.3 mm for canines (NobelActiveTM, Nobel Biocare, Gothenburg, Sweden) respectively, which were commonly used values for anterior mandibular teeth [27].

A selected dental implant was virtually placed along the long axis of the investigated tooth root with 4 mm of implant anchorage into native bone that was considered the minimum necessary to achieve primary stability [28, 29]. After placing the suitable implant virtually, labial bone perforation (LBP) or non-perforation were determined. The LBP was defined when the virtual implant extruded out of the apical outline of the labial cortical bone in the cross-sectioned and axial-viewed images (Supplemental Figure 3A) [14]. On the contrary, non-perforation was defined as the virtual implant within the apical outline of the labial cortical bone (Supplemental Figure 3B).

### Qualification and examination of CBCT images

All selected CBCT images of 1920 × 1080 pixel resolution, displayed on a 19-inch liquid-crystal display monitor (ChiMei Innolux Corporation, Taiwan), were examined by a commercially available three-dimensional (3D) navigation software (ImplantMax® 4.0; Saturn Image, Taipei, Taiwan) in a dimly lit environment. To ensure data reliability and reproducibility, all images were re-oriented so that the morphology of the crown and root, from cementoenamel junction (CEJ) to the apex, in the sagittal planes could be investigated undoubtedly as described previously (Supplemental Figure 2B) [22].

### Calibration and reliability between intra- and inter-examiners

The CBCT images were carefully inspected to follow the eligibility criteria by two independent investigators (first and corresponding author) twice, 1 week apart. Prior to the study, intra- and inter-examiner calibrations were performed on 50 randomly selective images based on the diagnosis of anatomic landmarks and anatomical measurements from CBCT images to assess data reliability. The nominal variables (e.g., perforation vs. non-perforation, CRDAP classification, Supplemental Table 2) and continuous variables (e.g., concavity depth, torque, dBT (0, mm), dBT (5, mm), dBT (10, mm)) and calibrated measurement errors, Cronbach’s alpha, and ICC (intraclass correlation coefficient) between intra- and inter-observations were also summarized (Supplemental Table 3). The Kappa statistic values for CRDAP and labial bone perforation were 0.940 and 0.929 for intra-observer agreement, and 0.929 and 0.932 for inter-observer agreement, respectively (Supplemental Table 2). Furthermore, the measurement errors, intraclass correlation coefficient, and Cronbach *α* values were also performed to confirm the reliability of intra- and inter-observations for continuous variable measurement (Supplemental Table 3). After calibration, the two independent investigators (first and corresponding author) evaluated the images separately, and any disagreement in image interpretation was discussed until a consensus was reached.

### Statistical analysis

The occurrences of LBP were expressed as the percentage of the number of sites with perforation divided by the total number of corresponding investigated sites. Pearson’s chi-square tests were used to examine differences with categorical variables, such as the frequency distribution of four types of CRDAP classification and the LBP of the investigated tooth in the anterior mandibular zone. Shapiro-Wilk test was used for testing the normality of data (data not shown). To compare the values of CD, CA, T, and deep bone thickness at perforation and non-perforation sites, Mann-Whitney *U* tests were performed for the non-normality. After multi-collinearity tests were performed to examine the linearity between each two variables, dBT (5) was removed from final regression analysis (data not shown). For analyzing the risk of LBP, multivariable logistic regression analysis with generalized estimating equation (GEE) method was used to handle repeated measurements of tooth sites in each subject which simultaneously adjusted for within factors of person and tooth sites, categorical (i.e., gender, tooth type, and crestal and radicular dentoalveolar phenotype (CRDAP)) and continuous (i.e., age, cavity depth (CD), cavity angle (CA), torque (T), and deep bone thickness (dBT)) variables. Four models were applied where Model 1 was adjusted for within factors (as our univariate model), Model 2 was adjusted for within factors, gender, and age, Model 3 was adjusted for within factors, gender, age, CD, CA, T, dBT (0), and dBT (10), and Model 4 was adjusted for within factors, gender, age, CA, CD, T, dBT (0), dBT (10), and CRDAP (CRDAP class III teeth were excluded due to no “perforation” teeth in this group). These adjusted variables in sequential models were determined to follow a theme from within factors, subject’s factors, and then anatomic factors influencing labial bone perforation with evidences proven by previous studies. All statistical analyses were performed by SPSS for Windows (PASW Statistics, version 18.0, SPSS, Inc., Chicago, IL, USA) and the level of statistical significance was set at *p* < 0.05.

## Results

### Qualified participants and CRDAP distribution

A total of 149 participants (894 teeth) retrieved from database met the inclusion criteria. The frequency distribution of CRDAP classification of investigated teeth was class IV (60.0%), followed by class II (18.8%), class I (10.9%), and class III (10.4%) (Table 1). Among the CRDAP class IV, canine teeth (70.5%) had the highest prevalence, compared with lateral incisors (56.4%) and central incisors (53.0%) (Table 1).

**Table 1:** Frequency distribution of four types of crestal and radicular dentoalveolar phenotype (CRDAP) of investigated teeth in the anterior mandibular region

	Total	CRDAP Class I		CRDAP Class II		CRDAP Class III		CRDAP Class IV		
Variables	*n*	*n*	%	*n*	%	*n*	%	*n*	%	*p*
Side										0.487
Right	447	44	9.8	92	20.6	46	10.3	265	59.3	
Left	447	53	11.9	76	17.0	47	10.5	271	60.6	
Tooth type										< 0.001
Central incisor	298	58	19.5	56	18.8	26	8.7	158	53.0	
Lateral incisor	298	25	8.4	74	24.8	31	10.4	168	56.4	
Canine	298	14	4.7	38	12.8	36	12.1	210	70.5	
Side and tooth type										< 0.001
Central incisor, right	149	24	16.1	31	20.8	13	8.7	81	54.4	
Central incisor, left	149	34	22.8	25	16.8	13	8.7	77	51.7	
Lateral incisor, right	149	13	8.7	38	25.5	16	10.7	82	55.0	
Lateral incisor, left	149	12	8.1	36	24.2	15	10.1	86	57.7	
Canine, right	149	7	4.7	23	15.4	17	11.4	102	68.5	
Canine, left	149	7	4.7	15	10.1	19	12.8	108	72.5	
Total	894	97	10.9	168	18.8	93	10.4	536	60.0	

The classification of crestal and radicular dentoalveolar phenotype (CRDAP) of mandibular anterior teeth was categorized according to the thickness of dentoalveolar bone at both crestal and radicular zones [25]. The chi-square tests were used for comparing the distribution of CRDAP in different “side,” “tooth type,” and “side and tooth type,” respectively. The level of statistical significance was set at *p* < 0.05

### Frequency distribution of labial bone perforation (LBP)

The overall probability of LBP of all investigated teeth was 21.6% (193 teeth), and the LBP was most likely to occur in canines (85 teeth; 28.5%) when compared with central and lateral incisors (Table 2). When comparing the risk of LBP using the CRDAP classification, class II had the highest probability of LBP (29.2%) compared with other CRDAP classes (Table 2). The significant differences were observed between tooth type (*p* < 0.001) and CRDPA classification (*p* < 0.001) (Table 2).

**Table 2:** Frequency distribution of labial bone perforation of investigated teeth in the anterior mandibular region

	Perforation		Non-perforation		
Variables	*n*	%	*n*	%	*p*
Total	193	21.6	701	78.4	
Side					0.464
Right	101	22.6	346	77.4	
Left	92	20.6	355	79.4	
Tooth type					< 0.001
Central incisor	63	21.1	235	78.9	
Lateral incisor	45	15.1	253	84.9	
Canine	85	28.5	213	71.5	
Side and tooth type					0.002
Central incisor, right	38	25.5	111	74.5	
Central incisor, left	25	16.8	124	83.2	
Lateral incisor, right	21	14.1	128	85.9	
Lateral incisor, left	24	16.1	125	83.9	
Canine, right	42	28.2	107	71.8	
Canine, left	43	28.9	106	71.1	
Tooth-alveolar ridge (CRDAP)					< 0.001*
CRDAP class I	3	3.1	94	96.9	
CRDAP class II	49	29.2	119	70.8	
CRDAP class III	0	0.0	93	100.0	
CRDAP class IV	141	26.3	395	73.7	

The classification of crestal and radicular dentoalveolar phenotype (CRDAP) of mandibular anterior teeth was categorized according to the thickness of dentoalveolar bone at both crestal and radicular zones [25]. The “perforation” was defined as the virtual implant extruded out of the apical outline of the labial cortical bone, whereas “non-perforation” was defined as the virtual implant within outline of the labial cortical bone. The chi-square tests were used for examining the frequency distribution of labial bone perforation (“perforation” vs. “non-perforation”) in different “sides,” “tooth types,” and “side and tooth types,” respectively. *The Fisher’s exact test was used to examine the frequency distribution of labial bone perforation (“perforation” vs. “non-perforation”) in different “tooth-alveolar ridge (CRDAPs).” The level of statistical significance was set at *p* < 0.05

### Morphologic parameters related to LBP

The concavity depth (CD) and torque (T) in the perforation group were statistically higher than in the non-perforation group, whereas concavity angle (CA) was lower in the perforation group than in the non-perforation group (Table 3). All these values had a significant impact on the probability of LBP (all *p* < 0.001). The bone thickness increased gradually toward the apical region, from dBTs (0) (8.20 ± 2.02 mm), dBTs (5) (9.41 ± 2.04 mm), to dBTs (10) (10.84 ± 2.00 mm), but bone thickness was statistically lower in perforation group than non-perforation group at each measured level (*p* < 0.001) (Table 3).

**Table 3:** Comparisons of concavity depth (CD, mm), concavity angle (CA, degree), torque (degree), and deep bone thickness (dBT, mm) at different levels (0, 5, and 10 mm) in the “perforation” and “non-perforation” sites of investigated teeth in anterior mandibular region

	Total		Perforation		Non-perforation		
Variables	Mean (SD)	Median (IQR)	Mean (SD)	Median (IQR)	Mean (SD)	Median (IQR)	p*
Alveolar ridge							
Concavity depth (CD)	4.44 (1.96)	4.2 (3.0)	5.59 (1.93)	5.6 (2.9)	4.12 (1.85)	3.8 (2.6)	< 0.001
Cavity angle (CA)	144.95 (7.36)	145.4 (9.4)	140.53 (7.13)	142.1 (9.0)	146.17 (6.95)	146.7 (8.8)	< 0.001
Deep bone thickness							
dBT (0)	8.20 (2.02)	8.0 (2.7)	6.87 (1.67)	6.6 (2.6)	8.57 (1.95)	8.4 (2.8)	< 0.001
dBT (5)	9.41 (2.04)	9.3 (2.6)	8.01 (1.79)	8.0 (2.8)	9.80 (1.93)	9.7 (2.6)	< 0.001
dBT (10)	10.84 (2.00)	10.8 (2.5)	9.77 (1.96)	10.0 (2.0)	11.14 (1.90)	11.0 (2.6)	< 0.001
Tooth-alveolar ridge							
Torque (T)	161.40 (8.62)	161.9 (12.1)	166.29 (7.94)	167.4 (11.3)	160.05 (8.32)	160.8 (10.9)	< 0.001

The deep bone thickness (dBT) was measured at 0, 5, and 10 mm from tooth root apex and along the long axis of the mandible, presented as dBT (0), dBT (5), and dBT (10), respectively [26]. The “perforation” was defined as the virtual implant extruded out of the apical outline of the labial cortical bone, whereas “non-perforation” was defined as the virtual implant within outline of the labial cortical bone. *The Mann-Whitney *U* tests were used to examine the differences of CD, CA, T, and dBT at “perforation” and “non-perforation” sites. The level of statistical significance was set at *p* < 0.05

Abbreviation: *IQR* interquartile range, *SD* standard deviation

### Further evaluation with regression model, including tooth-, alveolar ridge-, and tooth-alveolar ridge-associated factors

A multivariable logistic regression analysis with generalized estimating equation method was used which simultaneously adjusted for categorical and continuous variables as previously described (Table 4). Placing virtually placed immediate implants at the canines were 6.31 times more likely to cause LBP when compared with the central incisors (reference group) (*P* < 0.01) (Table 4). The deep bone thickness at 0 mm (dBT (0)) significantly influenced the probability of LBP (*P* < 0.01), but not at 10 mm. The results also showed that when the tooth is classified as CRDAP class II or class IV, it is much more likely to have LBP when compared with the CRDAP class I (reference group), respectively (Table 4).

**Table 4:** Multiple logistic regression analysis of variables contributing to labial bone perforation during virtual implant placement

	Model 1 (*n* = 894)		Model 2 (*n* = 894)		Model 3 (*n* = 894)		Model 4 (*n* = 801)	
Variables	OR*, **	95% CI	Adjusted OR	95% CI	Adjusted OR	95% CI	Adjusted OR	95% CI
Tooth								
Central incisor	Referent		Referent		Referent		Referent	
Lateral incisor	0.66**	0.49–0.90	0.66**	0.48–0.90	0.41**	0.24–0.73	0.29**	0.16–0.53
Canine	1.49*	1.08–2.06	1.50*	1.08–2.08	11.67**	5.27–25.85	6.31**	2.85–13.97
Alveolar ridge								
Concavity depth (CD)	1.47**	1.27–1.70	1.49**	1.29–1.72	1.56**	1.26–1.94	1.85**	1.47–2.32
Cavity angle (CA)	0.90**	0.87–0.93	0.89**	0.86–0.92	0.85**	0.80–0.90	0.84**	0.79–0.89
Deep bone thickness								
dBT (0)	0.59**	0.51–0.67	0.58**	0.50–0.66	0.43**	0.34–0.55	0.46**	0.36–0.58
dBT (10)	0.68**	0.59–0.78	0.68**	0.59–0.77	1.03	0.84–1.28	1.03	0.81–1.31
Tooth-alveolar ridge								
Torque (T)	1.10**	1.06–1.14	1.10**	1.07–1.14	1.25**	1.18–1.34	1.28**	1.21–1.36
CRDAP classification								
CRDAP class I	–	–	–	–	–	–	Referent	
CRDAP class II	–	–	–	–	–	–	21.39**	3.04–150.23
CRDAP class III	–	–	–	–	–	–	–	–
CRDAP class IV	–	–	–	–	-	–	47.26**	7.00–319.22

The classification of crestal and radicular dentoalveolar phenotype (CRDAP) of mandibular anterior teeth was categorized according to the thickness of dentoalveolar bone at both crestal and radicular zones [25]. A multivariable logistic regression analysis with generalized estimating equation (GEEs) method was used and which was simultaneously adjusted for categorical (i.e., gender, tooth types, and crestal and radicular dentoalveolar phenotype (CRDAP)) and continuous (i.e., age, cavity depth (CD), cavity angle (CA), torque (T), and deep bone thickness (dBT) variables. * and ** indicate statistical differences as *p* < 0.05, *p* < 0.01

Model 1: adjusted for within factors

Model 2: adjusted for within factors, gender and age

Model 3: adjusted for within factors, gender, age, tooth types, CD, CA, T, dBT (0), and dBT (10)

Model 4: adjusted for within factors, gender, age, tooth types, CD, CA, T, dBT (0), dBT (10), and CRDAP (CRDAP class III teeth were excluded due to no “perforation” teeth in this group).

Abbreviation: *OR* odds ratio, *95% CI* 95% confidence interval

## Discussion

This research evaluated the anatomic phenotype of the anterior mandible using the CRDAP classification and demonstrated a high prevalence of thin labial bone plate. In addition, the alveolar morphologic structure, including concavity depth (CD), concavity angle (CA), and deep bone thickness (dBT), might increase the risk of labial bone plate perforation and complicate the outcome in immediate implant placement (IIP) over anterior mandibular region. Therefore, this study highlights the importance of preoperative evaluation using 3-dimensional CBCT images.

In this study, the CRDAP class IV (60.0%) was the most prevalent in the anterior mandible, indicating the facial bone wall in the crestal and radicular area of teeth were thin (Table 1). This is in line with previous studies which showed that in the majority of the examined teeth, a thin facial bone pattern (≤ 1 mm) is exhibited at different levels [24, 30], which may imply the high prevalence of CRDAP class IV (Table 1). Consequently, thin facial bone wall may require simultaneous bone augmentation procedures during immediate implant placement because substantial bone resorption following tooth extraction occurs at a thin facial bone thickness [31, 32]. This is also the first study to investigate the prevalence of CRDAP classifications in different tooth types, in which canines account for the majority at CRDAP class IV (70.5%, Table 1).

In regard to the rate of LBP, the risk of perforation is closely related to tooth type (*p* < 0.001) and CRDAP classification (*p* < 0.001) (Table 2). The canine site (28.5%) presented highest prevalence of LBP, followed by central incisor (21.1%), and lateral incisor (15.1%). In terms of CRDAP classification, similar prevalence of LBP occurs in CRDAP class II (29.2%) and class IV (26.3%) teeth. In a previous study using navigated flapless transmucosal approach to place inter-foraminal implant in edentulous mandibles, two implants (2.5%), both of canines, had to be removed immediately due to buccal bone perforation [20], which corresponds with our present results that LBP occurred most frequently at canine sites compared to other anterior mandibular teeth (Table 2). Although immediate implant placement has had a high cumulative success rate in the anterior mandible region, significant dimensional changes of the facial bone are to be expected after replacing a tooth with a dental implant [21]. Therefore, thorough evaluation of a potential implant site, including preoperative assessment of thin facial bone wall (i.e., canine or CRDAP class IV), can indicate sites which may require bone augmentation or guided bone regeneration procedures to allow for reconstruction of the hard tissue deficiency and adequate support for esthetically pleasing soft tissues [24, 33].

The information about these anatomic features contributing to the occurrence of LBP in the anterior mandible region is limited. In this study, the regional anatomical parameters, including CD, CA, T, and dBT (Table 3), significantly influence the occurrence of LBP, compared with the non-perforation sites. Moreover, perforation was 6.31 more likely to occur at canine sites than central incisors (reference group), and tooth-alveolar ridge relationship classified as CRDAP class II or class IV was much more likely to have LBP when compared with CRDAP class I (reference group) (Table 4). Thus, the specific tooth type and tooth-alveolar ridge relationship could have a significant impact on the occurrence of LBP (all *p* < 0.01, Table 4). Taken together, it is necessary to take these anatomical factors into consideration while performing comprehensive pre-surgical examination.

Clinically, the ideal immediate implant position is based on a restoration-driven treatment plan with precise 3-D positioning of the implant, rather than on the amount of available bone [34]. According to the results of the current study, the probability of LBP in anterior mandibular region is common (21.6%, Table 2). Therefore, based on favorable long-term outcome, the simultaneous guided bone regeneration or delayed implant placement should be considered when the implant sites were associated with inadequate bone volume or quality [31, 32].

The prevailing concept for pre-surgical treatment plan should consist of precise 3-D positioning of the implant and guided implant surgery for facilitating a more predictable treatment outcome, and improve patient safety [35, 36]. Specifically, by reducing probability of LBP, surgeons are highly suggested to perform meticulous preoperative examinations and continuously re-evaluate the decision using 3-D CBCT analysis before placing dental implant into extraction placement immediately [26, 35, 36].

There are certain concerns when applying the findings of this study to clinical scenarios. First, computer-guided implant planning has proven to improve the predictability of the treatment goals and risk management [35]; however, further evaluations regarding the accuracy in transferring the virtual designed implant position intra-orally are still needed. Second, to prevent the occurrence of LBP, the long axis and position of implant may be adjusted or shifted lingually in clinical setting, which may incidentally damage the lingual arteries in anterior mandibular region, causing massive hemorrhage and even life-threatening events [5, 8]. Moreover, with the limitation of study design, the subjects’ general medical information (diabetes, osteoporosis, arthritis, immune status, etc.) and the cause of tooth loss could not be evaluated. In this study, subjects with severe alveolar destruction or obvious periodontal destruction were excluded to minimize the possibilities of subject-related factors [37, 38]. Concerning the measurement errors from CBCT, previous studies showed the mean absolute errors between CBCT and direct measurements appear to be limited and are unlikely to be clinically relevant with proper preliminary calibration [39]. The images with poorer quality, which resulted from minor patient movements during imaging which might contribute to measurement discrepancy between the image dimensions and realistic morphologies, were also excluded [40]. The current results regarding the prevalence of associated anatomical factors (i.e., CRDAP, CA, CD, T, dBT) show significant contributions to the occurrence of LBP while placing immediate implant in anterior mandible region. These results would provide further referent information, which constructs a preoperative automatic knowledge-based algorithm in CBCT image to assess risk in immediate implant surgery with identified important anatomic factors.

## Conclusion

To reduce the probability of LBP, meticulous preoperative assessments on anatomical parameters are highly suggested when placing dental implant immediately in the anterior mandible region.

## Supplementary Information

**Additional file 1: Supplemental Figure 1** (A) The flowchart of subject and image recruitment protocol. Subjects for full edentulous ridge, dental misalignment, alveolar bone destruction, and distorted image were excluded. (B) Images for tooth missing or implant replacement, pathologic lesion, and prosthodontic treatment were excluded. **Supplemental Figure 2** (A) A schematic description of the image orientation procedure. (B) The virtual implant position was verified mesiodistally (upper panel) and buccolingually (lower left panel) from reconstructed 3-dimensional images (lower right panel). **Supplemental Figure 3** Definition of labial bone perforation (LBP) in the anterior mandibular region while performing virtual immediate implant placement. (A) The perforation was defined when the virtual implant extruded out of the outline of the labial cortical bone in the cross-sectioned and axial-viewed images. (B) The non-perforation was defined as the virtual implant within the apical outline of the labial cortical bone.

**Additional file 2.**

## Data Availability

The datasets of the current study are available from the corresponding author on reasonable request.

## Abbreviations

CRDAP: Crestal and radicular dentoalveolar bone phenotype
CBCT: Cone beam computed tomography
DICOM: Digital imaging and communications in medicine
LBP: Labial bone perforation
CD: Concavity depth
CA: Concavity angle
T: Torque
dBT: Deep bone thickness
GEE: Generalized estimating equation
