# Supplementary material for: Risk assessment of labial bone perforation in the anterior mandibular region: a virtual immediate implant placement study
Source: Int J Implant Dent. 2021 Jul 26;7:68. doi: 10.1186/s40729-021-00351-w (PMC8310813; doi:10.1186/s40729-021-00351-w)
Supplement: Supplementary file 1 — Additional file 1: Supplemental Figure 1 (A) The flowchart of subject and image recruitment protocol. Subjects for full edentulous ridge, dental misalignment, alveolar bone destruction, and distorted image were excluded. (B) Images for tooth missing or implant replacement, pathologic lesion, and prosthodontic treatment were excluded. Supplemental Figure 2 (A) A schematic description of the image orientation procedure. (B) The virtual implant position was verified mesiodistally (upper panel) and buccolingually (lower left panel) from reconstructed 3-dimensional images (lower right panel). Supplemental Figure 3 Definition of labial bone perforation (LBP) in the anterior mandibular region while performing virtual immediate implant placement. (A) The perforation was defined when the virtual implant extruded out of the outline of the labial cortical bone in the cross-sectioned and axial-viewed images. (B) The non-perforation was defined as the virtual implant within the apical outline of the labial cortical bone. [file 40729_2021_351_MOESM1_ESM.docx]

**Supplemental Tables**

**Supplemental Table 1** Demographic characteristics of qualified subjects.

|  | **Male** |  | **Female** |  | **Total** |
| --- | --- | --- | --- | --- | --- |
| **Subject Variables** | n = 63 |  | n = 86 |  | n = 149 |
| Percentage (%) | 42.3% |  | 57.7% |  | 100% |
| Age (Mean±SD) | 50.6 ± 12.36 |  | 46.85 ± 13.31 |  | 48.44 ± 13.00 |
| Age range (Max-Min) | 25-85 |  | 20-76 |  | 20-85 |

**Supplemental Tables**

**Supplemental Table 2** Reliability of intra- and inter- observations for nominal variables measurement.

| **Nominal variables** | **disagreement** |  |  |  |
| --- | --- | --- | --- | --- |
| Intra-observation | Frequency |  | kappa |  |
| Perforation (Yes vs. No) | 1/50 |  | 0.940 |  |
| CRDAP classification (Class I, II, III, IV) | 2/50 |  | 0.929 |  |
| Inter-observation |  |  |  |  |
| Perforation (Yes vs. No) | 1/50 |  | 0.929 |  |
| CRDAP classification (Class I, II, III, IV) | 2/50 |  | 0.932 |  |

The classification of crestal and radicular dentoalveolar phenotype (CRDAP) of mandibular anterior teeth were categorized according to the thickness of dentoalveolar bone at both crestal and radicular zones. The “Perforation” was defined as the virtual implant extruded out of the apical outline of the labial cortical bone, whereas “Non-perforation” was defined as the virtual implant within outline of the labial cortical bone [14]. The Kappa analysis was performed to evaluate the reliability of nominal variables measurement.

**Supplemental Tables**

**Supplemental Table 3** Reliability of intra- and inter- observations for continuous variables measurement.

| **Continuous variables** |  |  |  |  |  |  |  |  |  |  |  |  |
| --- | --- | --- | --- | --- | --- | --- | --- | --- | --- | --- | --- | --- |
| Intra-observation | First observation | |  | Second observation | |  | Measurement errors | |  | ICC |  | Cronbach's Alpha |
|  | Mean | SD |  | Mean | SD |  | Mean | SD |  |  |  |  |
| Concavity depth (mm) | 6.93 | 2.76 |  | 6.93 | 2.76 |  | 0.12 | 0.10 |  | 0.998 |  | 0.999 |
| Concavity angle (degree) | 143.17 | 6.98 |  | 143.08 | 7.03 |  | 0.84 | 0.82 |  | 0.986 |  | 0.993 |
| Torque (degree) | 157.97 | 5.11 |  | 158.06 | 5.18 |  | 0.47 | 0.33 |  | 0.994 |  | 0.997 |
| dBT (0, mm) | 9.06 | 1.91 |  | 9.05 | 1.89 |  | 0.17 | 0.11 |  | 0.994 |  | 0.997 |
| dBT (5, mm) | 9.89 | 1.84 |  | 9.92 | 1.88 |  | 0.20 | 0.19 |  | 0.989 |  | 0.994 |
| dBT (10, mm) | 10.80 | 2.12 |  | 10.82 | 2.15 |  | 0.20 | 0.18 |  | 0.992 |  | 0.996 |
| Inter-observation | Observer 1 | |  | Observer 2 | |  | Measurement errors | |  | ICC |  | Cronbach's Alpha |
|  | Mean | SD |  | Mean | SD |  | Mean | SD |  |  |  |  |
| Concavity depth (mm) | 5.81 | 2.50 |  | 5.86 | 2.50 |  | 0.15 | 0.16 |  | 0.996 |  | 0.998 |
| Concavity angle (degree) | 144.18 | 6.22 |  | 143.99 | 6.35 |  | 0.89 | 0.75 |  | 0.983 |  | 0.992 |
| Torque (degree) | 158.68 | 4.95 |  | 158.94 | 5.15 |  | 0.58 | 0.44 |  | 0.991 |  | 0.995 |
| dBT (0, mm) | 8.98 | 2.06 |  | 8.99 | 2.00 |  | 0.24 | 0.23 |  | 0.986 |  | 0.993 |
| dBT (5, mm) | 9.98 | 1.97 |  | 10.10 | 1.95 |  | 0.22 | 0.22 |  | 0.989 |  | 0.994 |
| dBT (10, mm) | 11.06 | 2.16 |  | 11.10 | 2.16 |  | 0.23 | 0.21 |  | 0.989 |  | 0.995 |

Cronbach's Alpha was used for examining the reliability of continuous variables measurement.

Deep bone thickness, dBT; ICC, intraclass correlation coefficient; SD, standard derivation.
