# Supplementary figures and images for: Risk assessment of labial bone perforation in the anterior mandibular region: a virtual immediate implant placement study
Source: Int J Implant Dent. 2021 Jul 26;7:68. doi: 10.1186/s40729-021-00351-w (PMC8310813; doi:10.1186/s40729-021-00351-w)

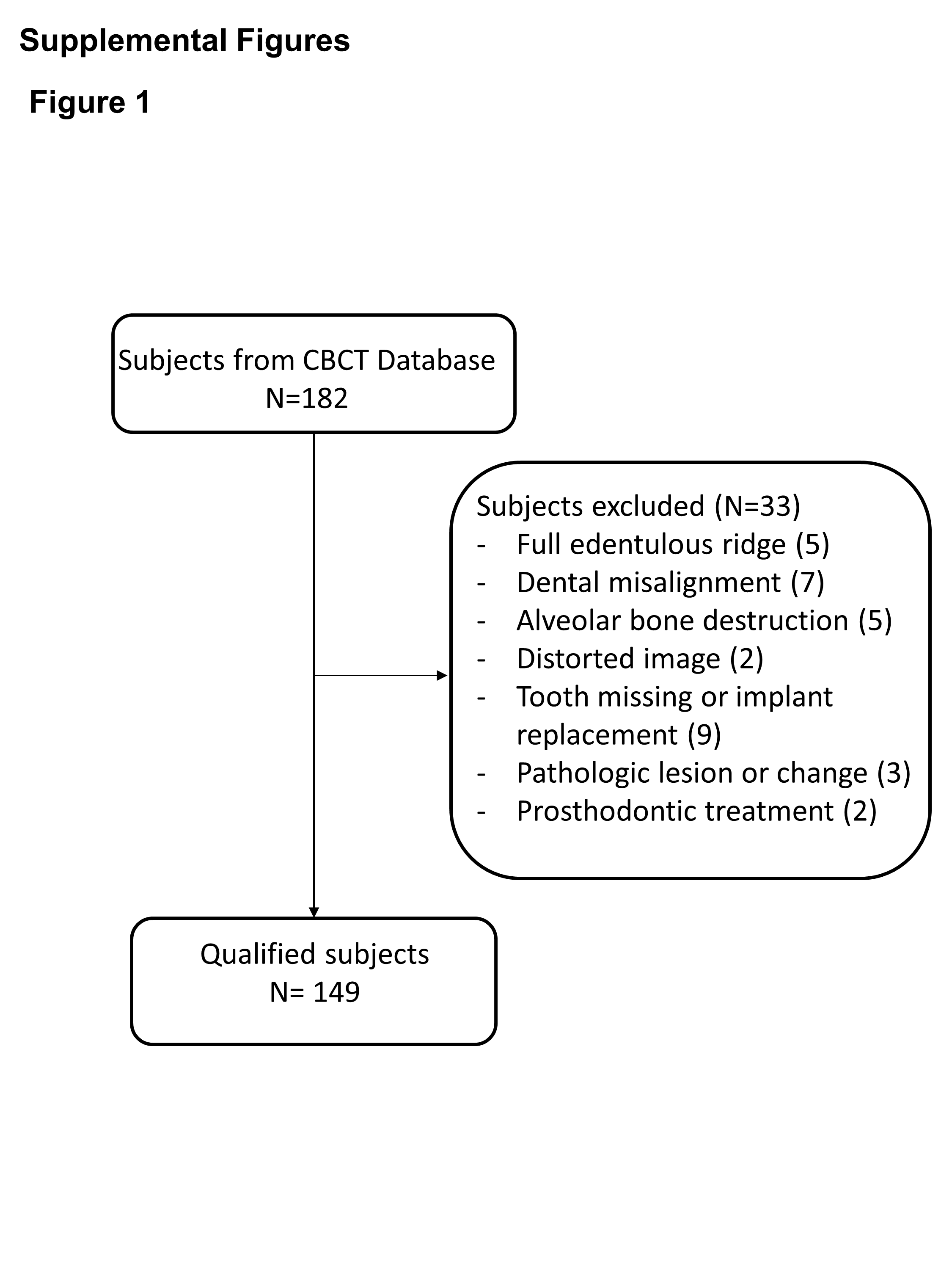

Supplement: Supplementary file 2 — Additional file 2. [file 40729_2021_351_MOESM2_ESM.zip › Fig. S1.TIF]

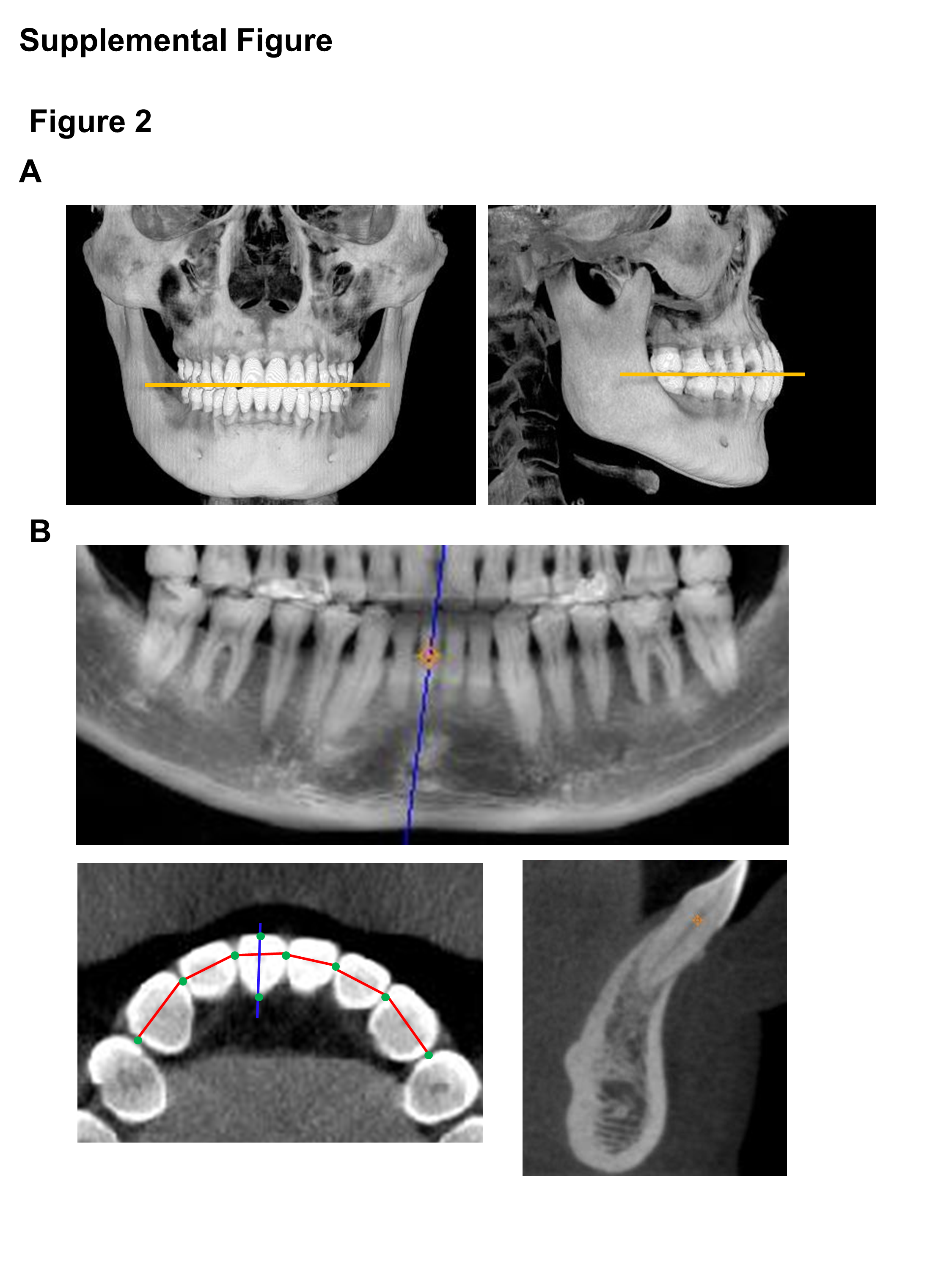

Supplement: Supplementary file 2 — Additional file 2. [file 40729_2021_351_MOESM2_ESM.zip › Fig. S2.TIF]

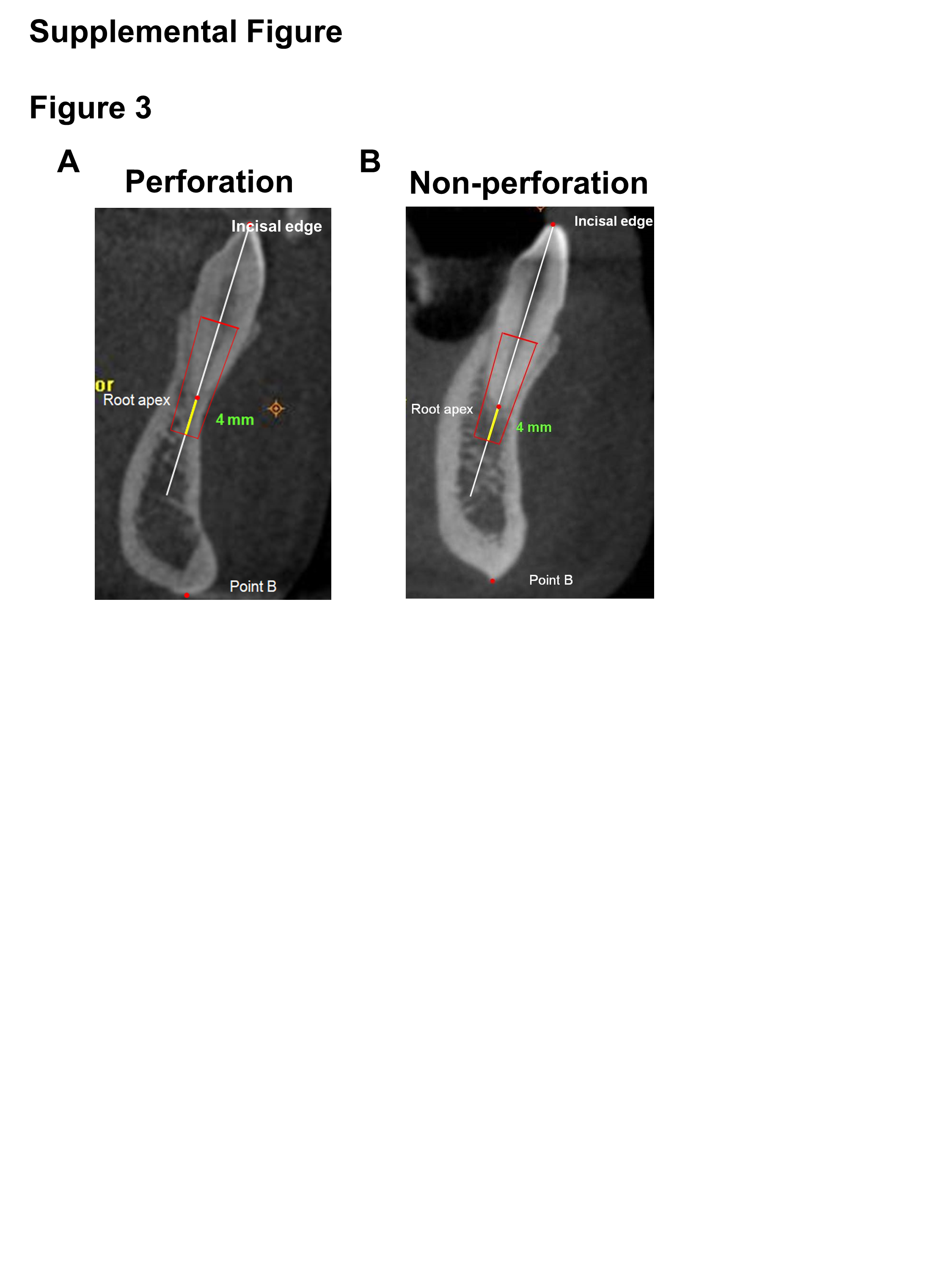

Supplement: Supplementary file 2 — Additional file 2. [file 40729_2021_351_MOESM2_ESM.zip › Fig. S3.TIF]
